# Supplementary material for: Humoral and cellular responses after a third dose of SARS-CoV-2 BNT162b2 vaccine in patients with lymphoid malignancies
Source: Nat Commun. 2022 Feb 14;13:864. doi: 10.1038/s41467-022-28578-0 (PMC8844396; doi:10.1038/s41467-022-28578-0)
Supplement: Supplementary file 1 — Supplementary Information [file 41467_2022_28578_MOESM1_ESM.pdf]

Supplementary Data file

Humoral and cellular responses after a third dose of SARS-CoV-2 BNT162b2 vaccine in patients with lymphoid malignancies.

Daniel Re (first author and corresponding author) and Jérôme Barrière (corresponding author)

Supplementary Figure 1

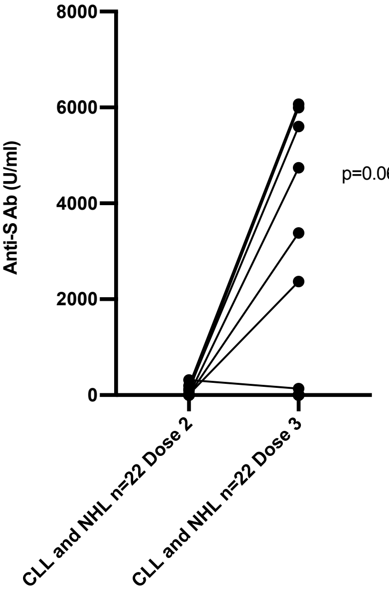

Supplementary Figure 1: Humoral response with Anti-S Abs titer. Shown is data of 22 patients with chronic lymphocytic leukemia and B-cell non-Hodgkin lymphoma after two and three doses of the BNT162b2 vaccine for whom IFN-γ secretion was studied (Wilcoxon matched-pairs signed rank test p=0.0547). Source data are provided as a Source Data file.

Supplementary Figure 2

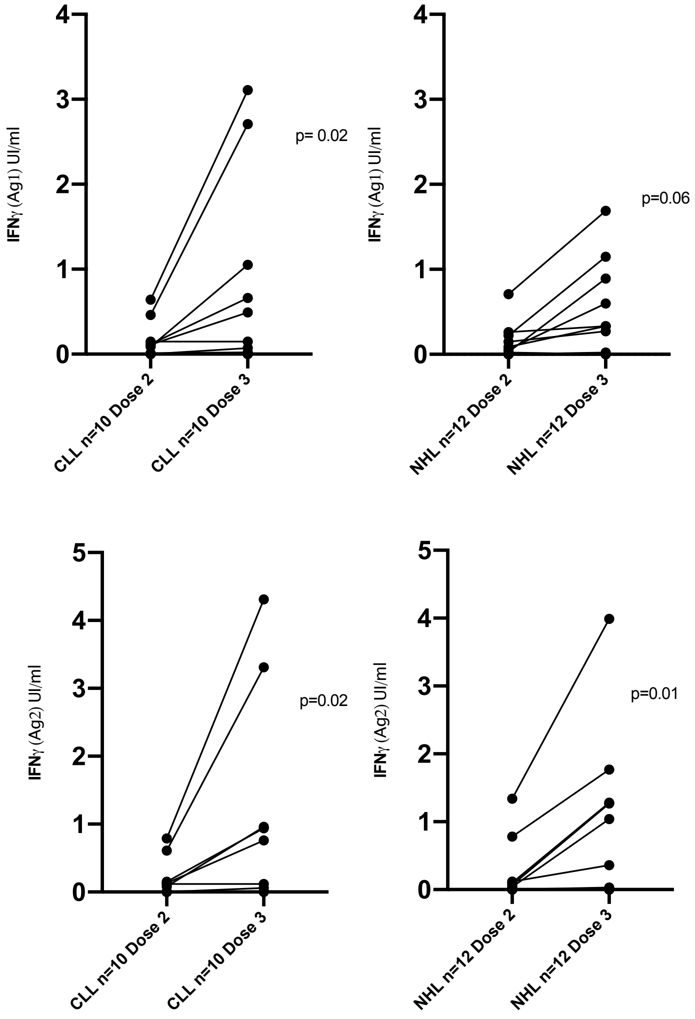

Supplementary Figure 2: Comparative IFN-γ secretion after exposition to antigen 1 or antigen 2 of SARS-CoV-2. Shown is data for 10 patients with chronic lymphocytic leukemia and 12 patients with B-cell non-Hodgkin lymphoma after a third dose of the BNT162b2 vaccine (Wicoxon matched-pairs signed rank test: CLL p=0.02 (antigen 1 and 2; NHL p=0.06 (antigen1) and p=0.01 (antigen 2)). Source data are provided as a Source Data file.

Supplementary Figure 3

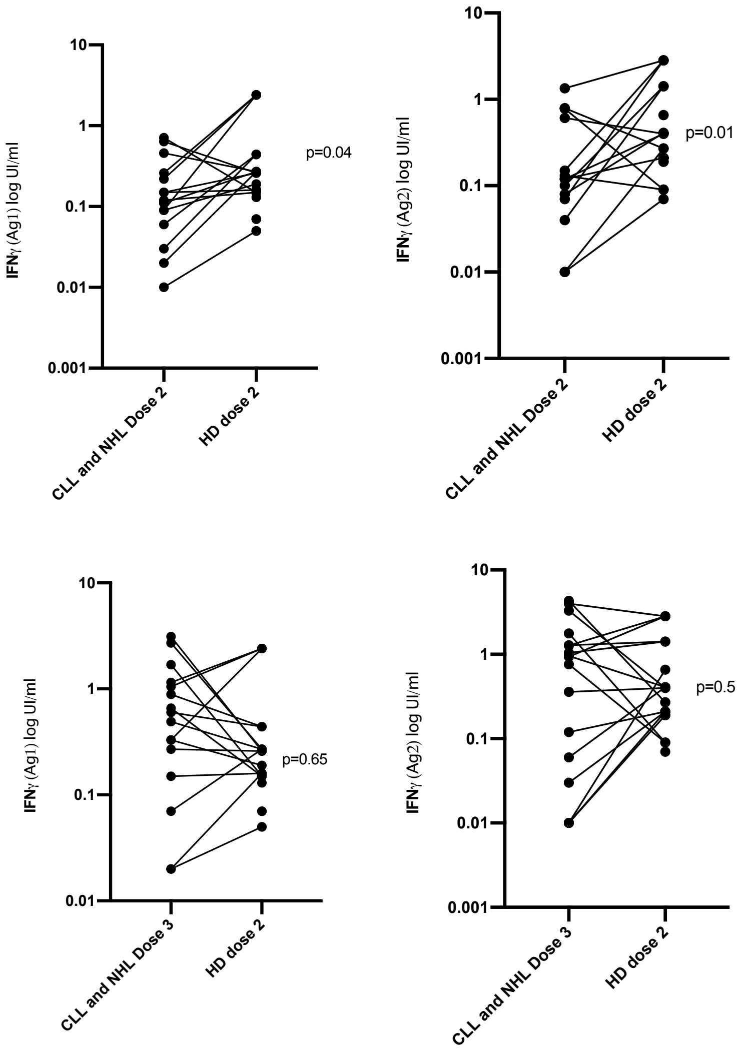

Supplementary Figure 3: Comparative IFN-γ secretion after exposition to antigen 1 or antigen 2 of SARS-CoV-2. Shown is IFN-γ secretion after exposition to antigen 1 or antigen 2 of SARS-CoV-2 in 22 patients with chronic lymphocytic leukemia and B-cell non-Hodgkin lymphoma after a second and third dose of the BNT162b2 vaccine compared to Healthy donors' controls (n=10) paired on age (by 10-year age groups) after two doses of the BNT162b2 vaccine (Wilcoxon matched-pairs signed rank test: after dose 2 p=0.04 (antigen1) and p=0.01 (antigen2)), after dose 3 (p=0.65 (antigen 1) and p=0.05 (antigen2)). Source data are provided as a Source Data.
