# Supplementary material for: Humoral and cellular responses after a third dose of SARS-CoV-2 BNT162b2 vaccine in patients with lymphoid malignancies
Source: Nat Commun. 2022 Feb 14;13:864. doi: 10.1038/s41467-022-28578-0 (PMC8844396; doi:10.1038/s41467-022-28578-0)
Supplement: Supplementary file 3 — Reporting Summary [file 41467_2022_28578_MOESM3_ESM.pdf]

nature portfolio

Corresponding author(s): Dr Daniel Re & Dr Jérôme Barrière

Last updated by author(s): Jan 15, 2022

Reporting Summary

Nature Portfolio wishes to improve the reproducibility of the work that we publish. This form provides structure for consistency and transparency in reporting. For further information on Nature Portfolio policies, see our Editorial Policies and the Editorial Policy Checklist.

Statistics

For all statistical analyses, confirm that the following items are present in the figure legend, table legend, main text, or Methods section.

n/a	Confirmed	
☐	☒	The exact sample size (*n*) for each experimental group/condition, given as a discrete number and unit of measurement
☐	☒	A statement on whether measurements were taken from distinct samples or whether the same sample was measured repeatedly
☐	☒	The statistical test(s) used AND whether they are one- or two-sided<br>*Only common tests should be described solely by name; describe more complex techniques in the Methods section.*
☐	☒	A description of all covariates tested
☐	☒	A description of any assumptions or corrections, such as tests of normality and adjustment for multiple comparisons
☐	☒	A full description of the statistical parameters including central tendency (e.g. means) or other basic estimates (e.g. regression coefficient) AND variation (e.g. standard deviation) or associated estimates of uncertainty (e.g. confidence intervals)
☐	☒	For null hypothesis testing, the test statistic (e.g. *F*, *t*, *r*) with confidence intervals, effect sizes, degrees of freedom and *P* value noted<br>*Give P values as exact values whenever suitable.*
☒	☐	For Bayesian analysis, information on the choice of priors and Markov chain Monte Carlo settings
☒	☐	For hierarchical and complex designs, identification of the appropriate level for tests and full reporting of outcomes
☒	☐	Estimates of effect sizes (e.g. Cohen's *d*, Pearson's *r*), indicating how they were calculated

Our web collection on statistics for biologists contains articles on many of the points above.

Software and code

Policy information about availability of computer code

Data collection	Excel MS
Data analysis	R.4.0.3 and GraphPad Prism software

For manuscripts utilizing custom algorithms or software that are central to the research but not yet described in published literature, software must be made available to editors and reviewers. We strongly encourage code deposition in a community repository (e.g. GitHub). See the Nature Portfolio guidelines for submitting code & software for further information.

Data

Policy information about availability of data

All manuscripts must include a data availability statement. This statement should provide the following information, where applicable:

- Accession codes, unique identifiers, or web links for publicly available datasets
- A description of any restrictions on data availability
- For clinical datasets or third party data, please ensure that the statement adheres to our policy

All data are included in the Supplemental Information or available from the authors upon reasonable requests, as are unique reagents used in this Article.

Field-specific reporting

Life sciences study design

All studies must disclose on these points even when the disclosure is negative.

Sample size	Observational study including a total of 45 patients with lymphoid malignancy (chronic lymphocytic leukemia, Non-Hodgkin lymphoma and multiple myeloma).
Data exclusions	Data sets of two patients were excluded as patients were in contact with Sars CoV2 (positivity of anti-N antibodies).
Replication	Findings were not replicated since data were obtained from routine biological assays.
Randomization	This is an observational non-randomized study.
Blinding	This is an observational non-randomized study, therefore no blinding was done.

Reporting for specific materials, systems and methods

We require information from authors about some types of materials, experimental systems and methods used in many studies. Here, indicate whether each material, system or method listed is relevant to your study. If you are not sure if a list item applies to your research, read the appropriate section before selecting a response.

Materials & experimental systems

n/a	Involved in the study	
☒	☐	Antibodies
☒	☐	Eukaryotic cell lines
☒	☐	Palaeontology and archaeology
☒	☐	Animals and other organisms
☐	☒	Human research participants
☐	☒	Clinical data
☒	☐	Dual use research of concern

Methods

n/a	Involved in the study	
☒	☐	ChIP-seq
☒	☐	Flow cytometry
☒	☐	MRI-based neuroimaging

Human research participants

Policy information about studies involving human research participants

Population characteristics	We analyzed a data set of 45 patients, prospectively included to receive dose 3 of the BNT162b2 vaccine given 78 days [range: 47-114] after dose 2 of the same vaccine. Included patients were suffering from chronic lymphocytic leukemia (CLL) (n=15), indolent and aggressive B-cell non-Hodgkin lymphoma (NHL) (n=14), and multiple myeloma (MM) (n=16). A cohort of 10 healthy donors' controls (HD) was also constituted (matched on sex ratio, median age 64 years old).
Recruitment	All eligible patients already participated in a previous observational study (see ref 9). A third dose of the BNT162b2 vaccine was administered on a voluntary basis to patients with lymphoid malignancy receiving active treatment or being at risk to require further treatment.
Ethics oversight	All participants signed a written informed consent and accepted their participation in this registered observatory in accordance with ethical and legal French policies that has been approved by our local ethics committee. The study design and conduct complied with all relevant regulations regarding the use of human study participants and was conducted in accordance with the criteria set by the Declaration of Helsinki.

Note that full information on the approval of the study protocol must also be provided in the manuscript.

Clinical data

Policy information about clinical studies

All manuscripts should comply with the ICMJEguidelines for publication of clinical research and a completedCONSORT checklist must be included with all submissions.

Clinical trial registration	Health Data Hub Registration number F20210324145532<br>https://www.health-data-hub.fr/projets/suivi-serologique-post-vaccination-sars-cov-2
Study protocol	A blood sample was drawn before and 3 to 4 weeks after the third dose of the vaccine. Serologic testing was done (1 ml whole blood) for the presence of anti N and anti S antibodies. In a second step, the presence of neutralizing antibodies were monitored. Cellular testing of T cell function was done using a SARS-CoV2 QuantiFERON assay (5 ml whole blood). The third dose of the vaccine was injected independently of the result of the immune testing. Side effects were assessed when the second blood sample is taken 3 to 4 weeks after administration of the third dose. The full protocol is available upon request (corresponding authors).

Data collection	Data was collected 4 weeks after the third booster dose using medical hospital charts.
Outcomes	Outcome was defined as serologic humoral (anti S antibodies and neutralizing antibodies) and cellular (QuantiFERON assay) response in patients vaccinated with dose 3.
